# RNA *N* ^6^-Methyladenosine in Cancer Metastasis: Roles, Mechanisms, and Applications

**DOI:** 10.3389/fonc.2021.681781

**Published:** 2021-06-15

**Authors:** Qin Dang, Bo Shao, Quanbo Zhou, Chen Chen, Yaxin Guo, Guixian Wang, Jinbo Liu, Quancheng Kan, Weitang Yuan, Zhenqiang Sun

**Affiliations:** ^1^ Department of Colorectal Surgery, The First Affiliated Hospital of Zhengzhou University, Zhengzhou, China; ^2^ Academy of Medical Sciences, Zhengzhou University, Zhengzhou, China; ^3^ School of Life Sciences, Zhengzhou University, Zhengzhou, China; ^4^ Department of Basic Medical, Academy of Medical Sciences of Zhengzhou University, Zhengzhou, China; ^5^ Henan Academy of Medical and Pharmaceutical Sciences, Zhengzhou University, Zhengzhou, China; ^6^ Department of Pharmacy, The First Affiliated Hospital of Zhengzhou University, Zhengzhou, China

**Keywords:** *N*^6^-methyladenosine methylation, cancer metastasis, non-coding RNAs, mRNAs, clinical applications

## Abstract

Cancer metastasis is a symptom of adverse prognosis, a prime origin of therapy failure, and a lethal challenge for cancer patients. *N* ^6^-methyladenosine (m^6^A), the most prevailing modification in messenger RNAs (mRNAs) and non-coding RNAs (ncRNAs) of higher eukaryotes, has attracted increasing attention. Growing studies have verified the pivotal roles of m^6^A methylation in controlling mRNAs and ncRNAs in diverse physiological processes. Remarkably, recent findings have showed that aberrant methylation of m^6^A-related RNAs could influence cancer metastasis. In this review, we illuminate how m^6^A modifiers act on mRNAs and ncRNAs and modulate metastasis in several cancers, and put forward the clinical application prospects of m^6^A methylation.

## Introduction

The discovery of RNA methylation modification, as well as the exploration of its chemical structure and biological function, have opened up a new field of epigenetic research (1–4). As the most pervasive RNA modification, m^6^A was extensively found in eukaryotes, prokaryotes and viruses (5–7). Foremost, the m^6^A locus contains consensus motif “RRACH” and is associated with splicing factors, transcript abundance and mRNA half-life (8). Regulated by methyltransferases, demethylases and binding proteins, m^6^A methylation is involved in the development of the skeletal system (9), nervous system (10), immune homeostasis (11), and pathological disease processes (12). Gene expression and biological functions various from the methylation levels of corresponding RNAs in distinct tissues, cell lines and research models (2, 13–16), such as the expression of *ZEB1 (* 17), *OCT4 (* 18), sex determining region Y-box2 (*SOX2*) (19), *HDGF* (20), and suppressor of cytokine signaling 2 (*SOCS2*) (21).

Distant and multiple organ metastasis seriously reduces the overall survival of cancer patients (22–25). Compelling clues have demonstrated that cancer metastasis involves multiple factors, such as gene mutations, cancer exosomes, cancer local microenvironment and immune selection (26–29). Further, epigenetic modification affects the development, early metastasis, treatment and prognosis of cancers (17, 30–35). RNA methylation provides us with a level of epigenetic regulation beyond that of DNA methylation and histone phosphorylation or acetylation. Therefore, m^6^A methylation is likely to provide approaches to study and restrain cancer metastasis. Currently, the mechanism of m^6^A methylation on cancer metastasis remains elusive. Herein, we reviewed the progress of m^6^A-related mRNAs and ncRNAs in cancer metastasis, supplemented the profound pathways between methylated RNAs and cancer metastasis, and proposed the clinical value of m^6^A members as cancer biomarkers and therapeutic targets.

## Detection of m^6^A Modification

M^6^A, one of the most ubiquitous epigenetic modification in eukaryotic RNA, plays profound roles in many biological development (36). Therefore, the detection of m^6^A is particularly significant for its functional research. Transcriptome-wide sequencing and high-sensitivity mass spectrometry were applied to map m^6^A modification sites and to detect and quantify modifications. Nanopore sequencing, a new and portable method to detect base modifications, along with well-characterized microbial references could serve as controls in the development and evaluation of future methods for the identification of base modifications from single-molecule sequencing data (37). To achieve rapid and accurate quantitative detection of m6A-RNA, some novel electrochemical immunosensors were invented (38–40). Additionally, Xiao et al. have found that m^6^A modifications preferentially occupy genes with CpG-rich promoters, features of which regulate RNA transcript m^6^A (5).

## m^6^A Members: Writer, Eraser, and Reader

**Figure 1:**
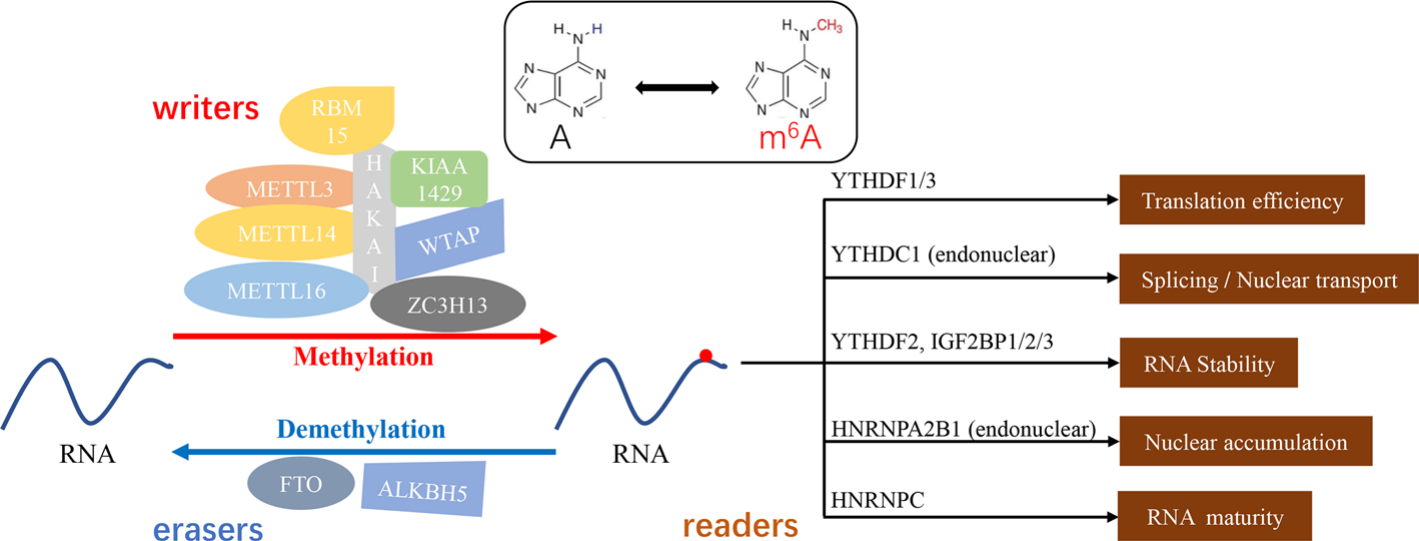
m^6^A methylation - a dynamic and reversible “chemical reaction”. “Writers” (METTL3/14/16, KIAA1429, WTAP, RBM15, ZC3H13, and HAKAI) or “erasers” (FTO, ALKBH5) mediate the process of methylation or demethylation. Furthermore, the function of modified substrates depends on the distinguishingly bounded “readers” (YTHDF1/2/3, YTHDC1, IGF2BP1/2/3, HNRNPA2B1, and HNRNPC).

The existing reports of methyltransferases are primarily focusing on methyltransferase-like 3 (METTL3), methyltransferase-like 14 (METTL14) and KIAA1429, which play a dominant role in the regulation between m^6^A methylation and cancer metastasis (17, 41–43). Studies have validated that METTL3 could participate in the fate determination of mRNA, resulting in the remarkable impact on the embryonic development, cell reprogramming, spermatogenesis, immune cell homeostasis, endothelial cell to hematopoietic cell transformation and so on (11, 44–49). As a synergistic protein of METTL3, METTL14 is not only involved in the biological processes mentioned above, but also attend the self-renewal and differentiation of embryonic stem cells and gametogenesis in mice (50). In cancer, METTL3-mediated methylation leads to epithelial mesenchymal transformation (EMT) and lung or liver metastasis in cancer patients (17, 19, 21, 51). Similar to METTL3, METTL14 was also proved to accelerate the progression of acute myeloid leukemia (AML) (52). KIAA1429 (VIRMA, via-like m^6^A methyltransferase associated) was found to be the largest molecule in the m^6^A methyltransferase complex (53). Silencing KIAA1429 has been reported to attenuate cell proliferation and liver cancer metastasis *in vitro* and *in vivo (* 54). In addition, Wilms’ tumor-associated protein (WTAP), RNA-binding motif protein 15(RBM15), Zinc finger CCCH domain containing protein 13(ZC3H13), HAKAI and methyltransferase-like 16 (METTL16) are also components of methyltransferase complex (43, 55–58) **(** **Figure 1** **)**.

Known as “erasers”, the demethylases could reverse the action of methyltransferases and make the m^6^A modification flexible and invertible (59). Fat mass and obesity-associated protein (FTO) as well as alkylation repair homing protein 5 (ALKBH5) are the two primary m^6^A erasers to be extensively studied. FTO has been implicated in weight gain and obesity and down-regulation of mRNA transcript levels. The aberrant expression of the FTO showed the enhancing effect on the chemoresistance and glioblastoma stem cell self-renewal (60–62). Niu et al. have proven that FTO could reduce apoptosis in breast cancer cells by inducing BNIP3 mRNA degradation (33). ALKBH5 is a key component in the proliferation and tumorigenesis of glioblastoma stem cells (63). ALKBH5 affects the tumorigenicity of human breast cancer cells and the spermatogenesis and fertility of mice (64, 65). Interestingly, ALKBH5 acts as a profound regulator in the maintenance and differentiation of cancer stem cells (CSCs), which is necessary for the formation and metastasis of primary cancers (65, 66). Moreover, FTO and ALKBH5 have been proposed to inhibit the EMT process. FTO is involved in the inhibition of EMT in intrahepatic cholangiocarcinoma (ICC) (53), and ALKBH5 impairs the migration and invasion of pancreatic cancer. Together, these m^6^A writers and erasers govern different states and processes of cancer metastasis.

Act as m^6^A readers, RNA-binding proteins have been evidenced to specifically select and bind to m^6^A sites (67, 68). YTHDC1 reads m^6^A sequences of mRNA and accelerates mRNA nuclear transport and alternative splicing (69). YTH *N* ^6^-methyladenosine RNA binding protein 1/3 (YTHDF1/YTHDF3) mediated methylation enhances the translation of mRNA. The binding of YTH *N* ^6^-methyladenosine RNA binding protein 2 (YTHDF2) to m^6^A site increases mRNA degradation (21, 70) **(** **Figure 1** **)**. What’s more, m^6^A methylation affects gene expression and cell function (12), such as maintaining the stability of genetic material, establishing epigenetic models, and mediating cellular and embryonic development (71–73).

Studies have shown that the inhibition of m^6^A-related enzymes leads to changes such as immune response, development of nervous system and blood system, indicating that m^6^A might be a significant modification in humans and interfere with the biological function (42, 74–76). Nevertheless, the specific mechanism remains to be further studied.

## Insights of m^6^A in Cancer Metastasis Mechanisms

**Figure 2:**
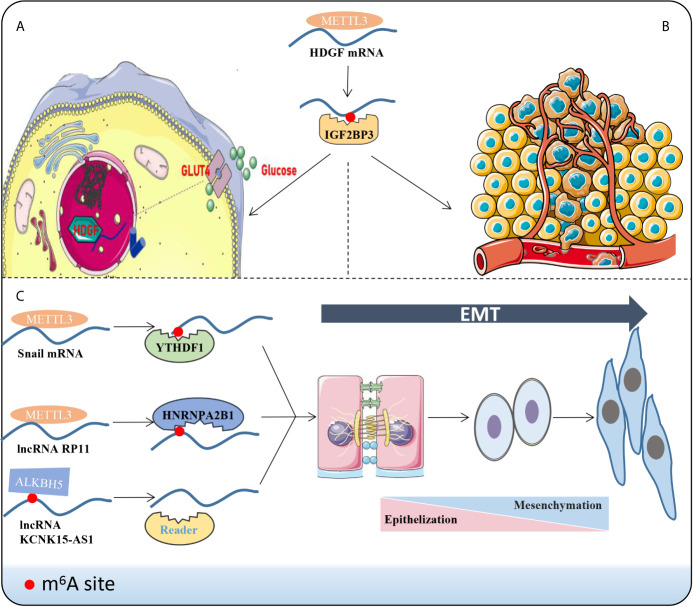
Phenotypic changes induced by m^6^A methylation. For instance, m^6^A reader IGF2BP3 combines the m^6^A sites to improve the stability of HDGF mRNA via METTL3 manner. Subsequently, HDGF accelerates the metabolism and glycolysis **(A)** of gastric cancer cells and promotes angiogenesis **(B)**. **(C)** LncRNAs or mRNAs bind to the corresponding “readers” and drive the EMT progression through the m^6^A manner.

Local infiltration and distant metastasis are the primary biological features of multiple malignancies and the leading cause of death (19, 77–79). Cancer metastasis refers to the process in which malignant cells spread to other parts to continue for growth after interacting with host cells, mainly including cell stemness formation (80), environmental angiogenesis or micro-angiogenesis (81), excessive glycolysis (82) and EMT transformation (83)**(** **Figure 2** **)**. Extensive findings suggest that m^6^A-related enzymes or proteins affect tumorigenesis (76), proliferation (84, 85), progression (86, 87) and metastasis (88, 89) in various mechanisms. Here, we emphasize the effects of different m^6^A-related molecules on cancer metastasis **(** **Table 1** **)**.

**Table 1:** Roles of key m^6^A members in the metastasis of various cancers.

Writer/Eraser	Roles	Tumor type	mRNA/ncRNA	Mechanism and pathway	Reader	Refs
METTL3	Oncogene	CRC	lnc RP11	Up-regulates RP11 nuclear accumulation, accelerates Siah1 and Fbxo45 mRNA degradation, prevents ZEB1 degradation, regulates EMT and enhance liver metastasis	HNRNPA2B1	(17)
			SOX2	Stabilizes SOX2 mRNA, induces CRC cell stemness, promotes drug resistance and lung metastasis	IGF2BP2	(19)
			pri-miR-1246	Promotes pri-miR-1246 maturation, down-regulates SPRED2, through MAPK pathway promotes phosphorylation	undetected	(90)
	Oncogene	HCC	SOCS2	Decreases SOCS2 mRNA stability, promotes chronic inflammation and lung metastasis	YTHDF2	(21)
			Snail	Activates the translation of Snail, promote EMT	YTHDF1	(60)
	Oncogene	GC	HDGF	Stabilize HDGF mRNA, then HDGF activate GLUT4 and ENO2 expression, accelerates angiogenesis and glycolysis, promotes liver metastasis	IGF2BP3	(20)
	Oncogene	NSCLC	①YAP ②lnc MALAT1	①Directly enhances YAP translation by recruiting YTHDF1/3 and eIF3b ②Stabilize MALAT1 mRNA, then MALAT1 sponges miR-1914-3p to promote metastasis through facilitating YAP activity	YTHDF3	(91)
	Oncogene	EOC	RHPN1-AS1	enhances RHPN1-AS1 transcriptional stability, promotes EOC cell viability and mobility	undetected	(89)
METTL14	Anti-oncogene	HCC	miR-126	Enhanced DGCR8 recognition of pri-miR126 and subsequent processing of mature miR-126	undetected	(92)
	Anti-oncogene	CRC	SOX4	Promote SOX4 mRNA degradation, suppress CRC metastasis through SOX4-mediated EMT process and PI3K/Akt signals	YTHDF2	(88)
KIAA1429	Oncogene	HCC	GATA3	Promote the degradation of GATA3 pre-mRNA, impairs the binding of HuR to GATA3 pre-mRNA	undetected	(54)
FTO	Oncogene	Breast cancer	BNIP3	Induces BNIP3 mRNA degradation, inhibit cell apoptosis and promote metastasis	YTHDF2	(33)
	Anti-oncogene	ICC	TEAD2	Impairs TEAD2 mRNA stability, promotes cisplatin-induced apoptosis, reduces angiogenesis	undetected	(53)
ALKBH5	Oncogene	Breast cancer	NANOG	Under hypoxia stimulation, increases translation of NANOG and enrichment of breast cancer stem cells	undetected	(65)
	Anti-oncogene	Pancreatic cancer	KCNK15-AS1	Upregulate KCNK15-AS1 expression, inhibits cell migration and invasion	undetected	(56)
	Anti-oncogene	NSCLC	YAP	Regulates the degradation or translation of YAP mRNA, decreased YAP activity by regulating miR-107/LATS2 axis in an HuR-dependent manner	YTHDF1/2/3	(93)

CRC, colorectal cancer; HCC, hepatocellular cancer; GC, gastric cancer; NSCLC, non-small-cell lung cancer; EOC, epithelial ovarian cancer; ICC, intrahepatic cholangiocarcinoma.

### m^6^A Methylation and EMT

EMT occurs in a variety of physiological and pathological conditions and is driven by a conservative set of induction signals, transcriptional regulators and downstream effectors (94). Cells exhibit enhanced fibroblast-like morphology and migration capability during EMT (94, 95). A recent report demonstrated that deletion of METTL3 impairs m^6^A and weakens the migration, invasion and EMT of hepatocellular carcinoma (HCC) cells *in vitro* and *in vivo (* 60). In colorectal cancer (CRC), a novel lncRNA named *RP11* is rich in m^6^A-RIP and regulated by METTL3 (17). The RP11/hnRNPA2B1 complex accelerates mRNA degradation of two E3 ligases, Siah1 (96) and Fbxo45 (97), and subsequently prevents proteasomal degradation of the key EMT-related transcription factor ZEB1 (17, 98). Chen et al. stated that METTL14 inhibits CRC malignant process partly through SOX4-mediated EMT and PI3K/Akt signaling (88). Additionally, m^6^A eraser ALKBH5 was down-regulated in pancreatic cancer cells, which could demethylate KCNK15-AS1 and regulate KCNK15-AS1-mediated cell motility and EMT process (56). In sum, m^6^A can indirectly facilitate or alleviate the extent of EMT by modifying EMT-related molecules. In order to improve current clinical strategies and to better predict the prognosis of patients, it seems evident that EMT is an indispensable aspect to be taken into account.

### m^6^A Methylation and Glycolysis

Recently, the field of tumor metabolism has received increasing attention. Aberrant metabolism is a biological sign of malignant tumors (81, 99, 100). Glycolysis has been widely validated to influence tumor progression and metastasis in HCC (81), BC (100), Oral squamous cell carcinoma (OSCC) (101) and leiomyosarcoma (102). Lin et al. validated that highly metastatic HCC cell lines display elevated glycolytic capacity (81). *HDGF* mRNA was detected to boost its stability by METTL3 methylation and bounding to m^6^A reader IGF2BP3 (20). Nuclear HDGF activates Glucose transporter-4 (*GLUT4*) and *ENO2* expression, followed by the acceleration of glycolysis in gastric cancer (GC) cells, which subsequently results in liver metastasis (20). Further, secretory HDGF released from the nucleus promotes cancer angiogenesis. The aerobic glycolysis genes, *GLUT4* and *ENO2*, also key glycolytic enzymes, have been reported in head and neck squamous cell carcinoma (103), pancreatic ductal carcinoma (104), and multiple myeloma (105). However, these glycolytic molecules have not been mentioned to be m^6^A modified and perform downstream functions in the head and neck tissue, the pancreatic duct tissue or bone marrow, and further studies are expected.

### m^6^A Methylation and Angiogenesis

Angiogenesis is an imbalance between pro-angiogenic factors and inhibitory factors, which leads to the activation and overproduction of the vascular system (106). Recently, reports of tumor growth through vascular selection (107) or without the formation of new angiogenesis (108) can be found in the literature. However, solid tumor growth with new blood vessels still dominates. Wang et al. investigated both tube formation and human umbilical vein endothelial cell (HUVEC) growth were significantly increased by overexpressing METTL3 compared with controls *in vitro (* 20). Similarly, A negative correlation was found between the expression of *FTO* and *CD34* in ICC (53). CD34, a tumor marker involved in angiogenesis, is used as a quantitative indicator of microvascular density (109). Hence, the expression of FTO was negatively correlated with local microvascular density (53). Mechanistically, FTO reduces the methylation level of *TEAD2* mRNA and impairs the stability of *TEAD2* mRNA and affects angiogenesis (53).

### m^6^A Methylation and Cancer Cell Stemness

Acknowledged as a group of cells capable of self-renewal, infinite proliferation and multidirectional differentiation (110), CSCs are the prime origins for the infinite growth of cancers, and the fundamental impetus for the recurrence, metastasis and drug resistance of malignant tumors (111). Through impressing the expression of CSCs related factors such as *SOX2 (* 112), *Oct4 (* 113) and *NANOG (* 65, 114), m^6^A modification of mRNA modulates embryonic stem cell pluripotency, and cancer metastasis, recurrence or treatment resistance can be considerably inhibited. Namely, METTL3 actuates CRC cell stemness *in vitro* by maintaining *SOX2* expression and inhibition of METTL3 was associated with markedly decreased CSC surface antigens such as CD133, CD44, and epithelial cell adhesion molecule (EpCAM) (19). Meanwhile, a decrease in sphere numbers and sizes as well as a strikingly reduced stem cell frequency were observed in METTL3-inhibited CRC cells (19). Exposed to hypoxia stimulated *ALKBH5* overexpression, which reduced the methylation of *NANOG* mRNA and increased the expression of *NANOG (* 65). Accordingly, the ability to generate clusters of daughter cells and the activity (115) of aldehyde dehydrogenase (ALDH) 1 activity (116) were significantly enhanced, which indicated the populations of breast cancer cells were enriched in BCSCs (65).

Together, the discovery of m^6^A in metastasis mechanism involves EMT, glycolysis, angiogenesis and cancer cell stemness, which may jointly provide distinguished indicators and drug intervention targets for the individualized diagnosis and medical care.

## Procedure of m^6^A on mRNAs and ncRNAs Throughout Metastasis

### m^6^A Embellishes mRNA and Is Involved in Cancer Metastasis

**Figure 3:**
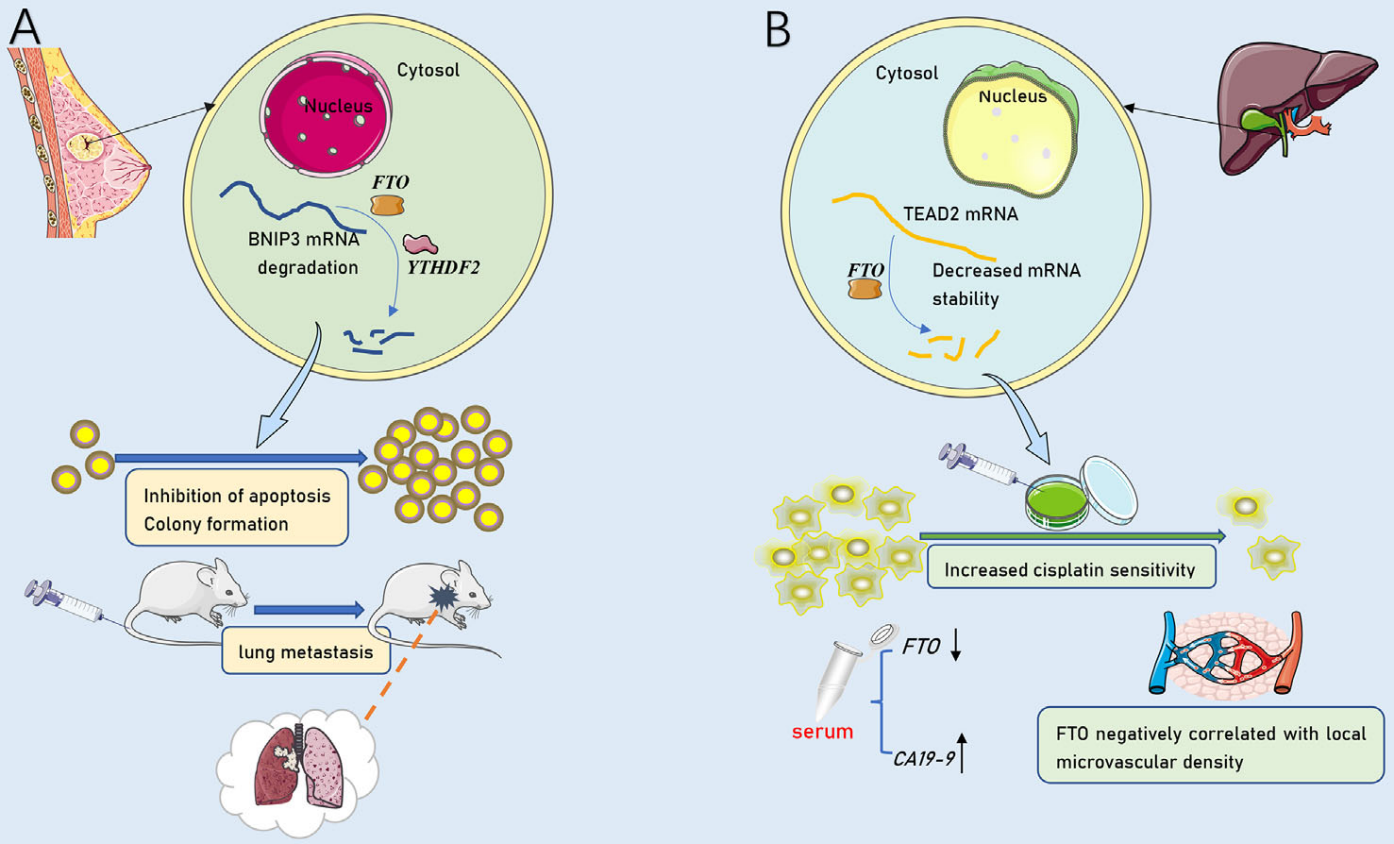
The dual role of FTO in tumor metastasis. **(A)** As an oncogenic molecule, FTO dramatically promoted breast cancer cell proliferation, colony formation and metastasis by down-regulating BNIP3. **(B)** As an anti-tumor molecule, FTO down-regulated TEAD2 mRNA stability and promoted cisplatin-induced ICC cell line apoptosis. FTO expression was negatively correlated with serum CA19-9 and local tumor microvascular density.

Extensive mRNA methylation allows epigenetic modifications to perform a broad scope of functions. Generally, METTL3, as an oncogene, catalyzes m^6^A methylation on mRNAs and facilitates cancers invasion, metastasis, and drug resistance (19–21, 60, 91). Instead, METTL14 primarily pose as an anti-oncogene and contribute to limiting tumor progression (88). As proof, METTL3 mediates insulin-like growth factor 2 mRNA binding protein 2 (IGF2BP2)-dependent SOX2 methylation to maintain the expression of *SOX2 (* 19, 19). The expression of genes in the core transcriptional regulatory network associated with *SOX2*, including cyclin D1 (*CCND1*) (117), Myc proto-oncogene protein (*MYC*) (118), and *POU5F1 (* 119), consistently decreased in METTL3-knockdown cells (19). Additionally, SOX2 can form transcription factor complexes with POU5F1. Collectively, METTL3 drives tumorigenesis, cell invasion and chemotherapy resistance of CRC cells might *via* METTL3/SOX2/CCND1-MYC-POU5F1 axis (19). The YTHDF2-dependent pathway makes METTL14 markedly enhanced *SOX4* mRNA m^6^A level and elevated mRNA degradation (88). METTL3 not only promotes HCC advancement through YTHDF2-dependent posttranscriptional silencing of *SOCS2 (* 21), but also facilitates CRC progression *via* IGF2BP2-dependent stabilizing of SOX2 mRNA (19). Moreover, METTL3 was involved in boosting the stability of *HDGF* mRNA by acts on the CDS region (60). Subsequently, m^6^A reader IGF2BP3 directly selects and binds to the m^6^A sites on *HDGF* mRNA and enhances stability. Evidence implies that the EMT transcription factor Snail can lead to the mass migration of squamous cell carcinoma (120). METTL3-mediated methylation activates *SNAIL* mRNA translation *via* binding to YTHDF1. Similarly, GATA3 is identified as a direct downstream target of KIAA1429-mediated m^6^A and *GATA3* precursor mRNA (pre-mRNA) serves as the substrate (54). *GATA3* pre-mRNA degradation is governed by m^6^A modification on GATA3 3’UTR actuated by KIAA1429 (54). GATA3, a member of the GATA transcription factor family, is a recently discovered key factor regulating cell differentiation and cytokine expression, promoting the development of Th2 cells and rendering the body in an immunosuppressed state (121). Interestingly, lncRNA *GATA3-AS* functions as a cis-acting element for KIAA1429 to interact with *GATA3* pre-mRNA (54). In addition, Exposure to hypoxia stimulates ALKBH5 expression, which leads to demethylation of *NANOG* mRNA capable of encoding pluripotent factors, increases *NANOG* translation, and advances the percentage of BCSCs (65). FTO mediates the demethylation of *BNIP3* mRNA and promotes self-degradation after binding with the YTHDF2 protein (33). FTO also serves as a cancer suppressor in ICC (53). The inhibition of FTO improves the stability of transcription-enhancing factor *TEAD2* mRNA (122), promotes cisplatin-induced apoptosis, and reduces angiogenesis in ICC cells (53). Notably, the function of m^6^A enzyme can be separate or even opposite in distinct cancers (**Figure 3**). For instance, FTO can not only accelerate the progression of AML (123) and breast cancer (33), but also be treated as an anti-oncogene in ICC (53). There might be more complicated regulatory networks involved in m^6^A demethylation-mediated regulation of cancer metastasis. Recently, a study proposed m^6^A on carRNAs can globally tune chromatin state and transcription, and METTL3 favors chromosome-associated regulatory RNAs (carRNAs) methylation (124). YTHDC1 facilitates decay of a subset of these m6A-modified carRNAs, including promoter-associated RNAs, enhancer RNAs and repeats RNAs, through the NEXT-mediated nuclear degradation (124). Therefore, m^6^A might have remarkable functions in the nucleus, which awaited thorough research.

### m^6^A Embellishes ncRNA and Is Involved in Cancer Metastasis

Although do not encode RNA, ncRNAs poses as gene monitor, facilitate gene expression (125), and participate in gene activation programs (126) at the levels of transcription, RNA processing and translation (127). Evidence showed that METTL3 heightens the nuclear accumulation of lncRNA RP11 and generates RP11 highly expressed in CRC tissues (17). Clinical analysis revealed that RP11 considerably upregulates the EMT-related transcription factor ZEB1 (98), triggers EMT and liver metastasis through the RP11/HNRNPA2B1/E3 ligase (Siah1and Fbxo45)/ZEB1 axis, and positively correlated with CRC stage in patients (17). Similarly, it is widely shared that YAP family has a hand in EMT, angiogenesis, and cell proliferation and apoptosis of a variety of cancers, such as CRC (70), non-small-cell lung cancer (NSCLC) (128), and Oral squamous cell carcinoma (OSCC) (129). METTL3/YTHDF3 complex increases the stability of lnc *MALAT1* in an m^6^A manner and MALAT1 sponges miR-1914-3p to promote *YAP-1* expression (91). There is another reported way by which METTL3 enhances the stability and translation of *YAP* mRNA in NSCLC. *YAP* mRNA is directly identified and combines with YTHDF1/3 and eukaryotic initiation factor 3b (eIF3b) to act on the translation initiation factor and improve the translation efficiency (91) **(** **Table 1** **)**. Aberrant expression of miRNA in related diseases is caused by the anomalous methylation of the bases in the promoter region of the miRNA gene (130, 131). Wen et al. argued that upregulated METTL3 promotes metastasis of CRC *via* methylates pri-miR-1246, which further promotes the maturation of pri-miR-1246, and the miR-1246/SPRED2/MAPK signaling pathway is involved (90). Another study revealed that pri-miR-126 was transformed into mature miR-126 in a DiGeorge syndrome chromosomal region 8 (DGCR8)-dependent and m^6^A methylation manner (92). Knockdown of ALKBH5 results in the acquisition of m^6^A and reduction the association with Human antigen R(HuR) on the Forkhead box protein M1(FOXM1) transcript. Interestingly, FOXM1AS accommodates the interplay between ALKBH5 and FOXM1 (63). Additionally, lncRNA *RHPN1-AS1*, which contains METTL3-mediated m^6^A information, may be the reason for the increased stability of *RHPN1-AS1* and high expression in epithelial ovarian cancer (EOC) (89). Since *RHPN1-AS1* acts as ceRNA to antagonize miR-596 and up-regulate LETM1, leading to the metastasis of EOC, METTL3 can be inferred to be the oncogene of EOC *via* m^6^A manner (132). In brief, m^6^A methylation makes sense in epigenetic modification by affecting the translation, stability, degradation and expression of key mRNAs and ncRNAs.

## m^6^A Mediated Metastasis in Cancers

### Digestive System Cancers

#### CRC

Accumulating studies have confirmed that CRC is no longer a single tumor type, and it is more accurate to describe it as a group of heterogeneous diseases due to genetic and epigenetic changes (133). These results led to an increase in CRC typing; the clinical effect of molecular targeted drugs is also unsatisfactory, especially in some patients with distant metastasis. Recently, the epigenetic characteristics of CRC metastasis to the liver and lung were described. METTL14 promotes cancer proliferation and metastasis by promoting the EMT, protein phosphorylation or stemness of cancer cells through downstream targets such as lncRNA RP11 and microRNAs, which greatly improves the distant organ metastasis ability of CRC (17, 19, 90). In addition, CSCs are considered to be the cause of chemotherapy resistance in CRC (134, 135). These studies indicate that m^6^A modification might provide new drug targets for the accurate therapy and early prevention of CRC metastasis.

#### HCC

Most HCC patients are in an advanced stage at the time of diagnosis and usually accompanied with metastasis (136). At present, combined therapies, including surgery, liver transplantation, interventional and targeted therapy, prolong the survival period of patients with advanced liver cancer to a certain extent (137). However, the 5-year survival rate of HCC patients leaves much to be desired (138). METTL3 impairs the stability of *SOCS2* mRNA and inhibits the chronic inflammation (21). Moreover, the imbalance of METTL3 promotes the occurrence of HCC and chemotherapy resistance by regulating the phosphorylation pathway of METTL3/SOCS2/STAT5, which refers to key anti-oncogenes at the post-transcriptional level (21). In addition, there is a correlation between the level of methylation modification and the prognosis of HCC patients. Moreover, methyltransferase KIAA1429 acts on the 3’-UTR of *GATA3* pre-mRNA, and the methylated modified RNA binds to HuR protein, which drives malignant phenotypes of HCC (54).

#### GC

Due to late diagnosis and distant metastasis, the quality of life in GC patients is seriously reduced (139). At present, it is urgent to explore a pleasurable diagnostic and prognostic marker and treatment target for GC. Recently, Wang et al. argued that m^6^A methylation mediated by METTL3 promotes the m^6^A modification of *HDGF* mRNA, and the m^6^A reader IGF2BP3 directly recognizes and combines the m^6^A site on *HDGF* mRNA to enhance the stability (51). HDGF increases glycolysis and angiogenesis in GC cells, which are involved in the progression and metastasis of GC. This suggests that *HDGF* mRNA methylation promotes GC growth and leads to poor prognosis. METTL3, as a carcinogen, might be a new biomarker and therapeutic target for GC.

#### Pancreatic Cancer

Pancreatic cancer is one of the deadliest cancers in the world. It is usually diagnosed in the terminal stage, and other parts of the body have been metastasized at the time of diagnosis, with poor prognosis (140, 141). At the level of epigenetic regulation, a study of DNA methylation and the stemness of pancreatic cancer cells confirmed the effect of methylation on the metastasis (140). Additionally, studies have shown that ALKBH5, a demethylase, inhibits the invasion and tissue transfer of pancreatic cancer by reducing the methylation level of lncRNA *KCNK15-AS1* (56). The expression level of lncRNA *KCNK15-AS1* is negatively correlated with its methylation level. However, the mechanism between the stability of lncRNA *KCNK15-AS1* and m^6^A methylation is elusive. These mechanisms need to be further studied to find valuable therapies to prevent and control pancreatic cancer.

#### ICC

ICC is a highly heterogeneous malignant type of HCC (142). Its early clinical symptoms are not easy to perceive, and there is no specific target for clinical detection or treatment (111). Similar to pancreatic cancer, the relationship between ICC and m^6^A methylation has been less reported. Rong et al. found that the protein level of FTO decreased in samples and cell lines of ICC patients. The expression of FTO was negatively correlated with clinical cancer metastasis-related factors, such as cancer local microvascular density and CA19-9 concentration in serum (53) (**Figure 3B**). Therefore, m^6^A methylation might provide a novel direction for the clinical strategies of ICC treatment.

### Female Reproductive System Cancers

#### Breast Cancer

Breast cancer is a malignant and invasive tumor that seriously endangers women’s health. It is considered to be the cause of death of approximately 23% of postmenopausal women and is a global problem (143). Although the expression and regulation patterns of target genes related to breast cancer have been extensively studied, little is known about the post-transcriptional regulation mechanisms of gene expression in breast cancer metastatic. The abnormal expression of hepatitis B x-interacting protein (*HBXIP*) drives the proliferation and metastasis of breast cancer. HBXIP upregulates METTL14 by inhibiting microRNA(miRNA) *let-7g*. However, METTL14 increases the expression of *HBXIP*, thus forming an HBXIP/let-7g/METTL14/HBXIP loop (144). More interestingly, some studies found that METTL14 improves its translation efficiency without changing the mRNA expression level; that is, the target mRNA abundance remains unchanged (145). Preceding studies suggested that FTO mediates changes in energy metabolism to regulate weight and growth in adults (146, 147). Recent studies found that the expression of FTO is upregulated in human breast cancer. The down-regulation of FTO, knockdown, the proliferation and metastasis of breast cancer tissues and cells were significantly inhibited, while the number of apoptotic cancer cells increased. Further studies confirmed that FTO-mediated m^6^A methylation acts on BNIP3 transcripts that translate apoptotic proteins. Then, YTHDF2 binds to the demethylated *BNIP3* mRNA to inhibit BNIP3-induced apoptosis by reducing the expression of *BNIP3 (* 33). Accordingly, Niu et al. found that the demethylase FTO acts as an oncogene and promotes the progression of breast cancer through the FTO/BNIP3/Bcl2 anti-apoptosis signaling pathway (33). The study also revealed that the silencing of FTO suppressed lung metastasis in female Balb/c mice. Subsequent studies demonstrated this in mouse models of lung metastasis (**Figure 3A**). Another study on ALKBH5 found that the exposure of breast cancer cells to hypoxia significantly increased the expression of ALKBH5 in tissues and cells. Then, the demethylation of pluripotent *NANOG* mRNA increased the expression of *NANOG* and induced the enrichment of CSCs (65).

#### EOC

Recently, lncRNAs have been found in the occurrence, development and metastasis of ovarian cancer (83, 148, 149). LncRNA *DNM3OS* acts as an accelerator in promoting EMT in ovarian cancer (150). A recent report denoted the possibility of *RHPN1-AS1* be used as a ceRNA in the METTL3/RHPN1-AS1/miR-596/LETM1 axis to upregulate the expression of *LETM1 (* 89). Subsequently, LETM1 activates the FAK/PI3K/Akt pathway and causes the migration and invasion of EOC cells. The regulation of lncRNA *RHPN1-AS1* by m^6^A modification may provide clues for the discovery of promising diagnostic markers or drug therapeutic targets for EOC patients.

### Respiratory System Cancers

#### NSCLC

Due to the limitations of clinical treatment, there are still no better treatment measures to limit the progression and metastasis of lung cancer. Interestingly, the YAP pathway was reported to promote drug resistance, progression and metastasis of NSCLC (9, 128). *YAP* expression is negatively correlated with ALKBH5 expression and serves as an opposite role in the regulation of cellular proliferation, invasion, migration, and EMT of NSCLC cells (93). ALKBH5 impaired cancer growth and metastasis *in vivo* by decreasing the expression and activity of YAP (93). Meanwhile, the reduction of YAP m^6^A level by METTL3 knockdown inhibits NSCLC growth and enhances sensitivity to DDP *in vivo (* 91). The above studies indicate that METTL3 or ALKBH5 might be latent targets for inhibiting the progression and metastasis of NSCLC.

### OSCC

OSCC is the most common type of malignant tumor occurring in human oral cavity, with the highest degree of malignancy and the largest head and neck injury, and generally the worst prognosis (129, 151). METTL3-mediated m^6^A modifies the 3 ‘UTR region of *BMI1*, which is then recognized by IGF2BP1 and leads to up-regulation of *BMI1* translation (152). While, *BMI1* serves as an oncogene and targeting *BMI1* suppresses cancer growth and prevents relapse (153). Under the catalysis of METTL3, hypermethylation levels promoted the proliferation, self-renewal, migration and invasion of OSCC cells *in vitro*. Hypermethylation have also been proven to attend lung and popliteal lymph node metastasis of OSCC *in vivo*.

#### Nasopharyngeal Carcinoma

Nasopharyngeal carcinoma (NPC) is an endemic disease associated with Epstein-Barr virus infection, genetic element and environmental factors in Southeast Asian countries, and is also a high incidence in southern China (154, 155). Easy to relapse and early metastasis are the important barriers in nasopharyngeal carcinoma treatment. In analyzing differentially expressed m^6^A-related genes in 55 NPC patients and 20 control patients, Lu et al. found that upregulation of IGF2BP1 and downregulation of METTL3 were associated with poorer progress-free survival in NPC patients (156). Consensus cluster analysis and risk model predict that METTL3 is a risk factor for NPC metastasis. Additionally, immunohistochemical technique successfully verified the difference of METTL3 expression in NPC tissues (156). Thereby, m^6^A methylation mechanism may be a promising therapeutic target for NPC.

## Potential Clinical Applications

Theoretically, m^6^A methylation regulators are posed as efficacious pharmacological targets for anti-cancer drug in solving clinical problems. To illustrate, METTL3 gives rise to unfavorable prognosis by maintaining the stable expression of SOX2 in CRC and glioma (19, 157). Similarly, METTL3 activates the transcription regulator Snail translation and promotes EMT by acting on the mRNA coding region (60). A higher METTL3 expression level was positively associated with advanced OSCC stage and poor 5-year overall survival (152). In addition, METTL3 induces resistance to DDP and metastasis by increasing the extent of m^6^A methylation of YAP *in vivo (* 91). In brief, reliable data have suggested that METTL3 might be used as a prognostic indicator or as a reference item for diagnosing early metastasis of cancers. Likewise, both the univariate and multivariate Cox regression analysis were indicative of METTL14 was an independent prognostic factor in CRC (88). Moreover, METTL14 acts as one of the indexes reflecting the recurrence-free survival of HCC and the absence of METTL14 is related to the metastasis *in vitro* and *in vivo (* 92). Interestingly, METTL14 was responsible for the aberrant methylation modification in HCC, not METTL3 (92).

As previously mentioned, CSCs are considered to be the cause of chemotherapy resistance in CRC (134, 135). SOX2 is a marker of CSCs and has strong carcinogenic and metastatic potential. SOX2 guides stem cell formation and drug resistance in pancreatic cancer and bladder cancer (158). Markedly, Rhein could reversibly bind to FTO catalytic domain and competitively prevent the recognition of m^6^A modification substrates or alleviated the growth of subcutaneous breast cancer in mice, while another FTO inhibitor meclofenamic acid could effectively increase mRNA methylation levels in glioblastoma stem cells (GSCs) and suppress GSC growth (33, 159). Mechanistically, meclofenamic acid is a non-steroidal anti-inflammatory drug that competes with FTO to bind to RNA substrates containing m^6^A modification sites (159). Additionally, the exploration of distinguished FTO inhibitors is considered to be the preferrable treatment strategy for BC (160). Collectively, these FTO inhibitors were rarely verified in the human body or in clinical trials. Accordingly, it is of great significance to select appropriate molecular detection targets or specific drugs for early screening, diagnosis, therapeutic intervention and prognosis evaluation of patients in the future.

## Conclusion

To conclude, m^6^A methylation has a hand in diversified processes of cancer metastasis, such as facilitates EMT (17, 60, 88), sustains cancer cell stemness formation (19, 65), accelerates metabolism and glycolysis (21, 90), and favor angiogenesis (20). Strikingly, the dual effects of FTO (33, 53) and ALKBH5 (56, 65) published so far remind us that m^6^A manner pose as both a propellent and a restrainer in distinct cancers metastasis. Additionally, the presence of m^6^A-related regulators or pathways has been widely demonstrated in the metastasis of various solid tumors, such as digestive tumors (17, 20, 21, 56), female reproductive system tumors (33, 89), and respiratory system tumors (91, 152, 156). This is far from an isolated scenario, as m^6^A methylation regulates migration, invasion and drug resistance by targeting mRNAs and ncRNAs, which makes a profound contribution to the metastasis of malignant tumors. Moreover, with the gradual invention of means such as electrochemical immunoreceptors to detect m^6^A sites, m^6^A modification are likely to have further biological roles to be unearthed.

Given summarized above, the relationship between m^6^A methylation and cancer metastasis is further complicated and diverse than originally thought, as it cannot be readily concluded that m^6^A-related enzymes hold carcinogenic character only by increasing the extent of methylation modification and vice versa. Likewise, the knowledge of Rhein, the effective FTO inhibitor, competitively prevented the recognition of m^6^A modified substrate by FTO, which alleviated the growth of subcutaneous breast cancer in mice (33). Nevertheless, it is worth noting that the expected clinical drug trials related to m^6^A substitutes or inhibitors remain rarely carried out. m^6^A modulators, once lurking out of sight, are feasible to be used as sensitive biomarkers or effective intervention for early screening or individualized comprehensive therapy in cancer metastasis. Epigenetic regulation based on m^6^A methylation stands a good chance of opening up a novel dimension in cancer research.

## Author Contributions

ZS, WY, and QK provided direction and guidance throughout the preparation of this manuscript. QD wrote and edited the manuscript. BS, CC, and YG reviewed and made significant revisions to the manuscript. QZ, GW, and JL collected and prepared the related papers. All authors read and approved the final manuscript. All authors contributed to the article and approved the submitted version.

## Funding

This study was supported by The National Natural Science Foundation of China (81972663, 81560385), Key Scientific Research Projects of Institutions of Higher Education in Henan Province (19A310024), The Medical Scientific and Technological Research Project of Henan Province (201702027), Youth Innovation Fund Project of The First Affiliated Hospital of Zhengzhou University (YNQN2017035), The China Postdoctoral Science Foundation (2019T120648, 2017M610462), The National Natural Science Foundation of Henan Province (182300410342), and The Health Commission Technology Talents Overseas Training Project of Henan Province (2018140).

## Conflict of Interest

The authors declare that the research was conducted in the absence of any commercial or financial relationships that could be construed as a potential conflict of interest.

## Abbreviations

M^6^A, *N* ^6^-methyladenosine; M^1^A, *N* ^1^ atom of adenine; M^5^C,5-methylcytosine; M^7^G, *N* ^7^-methylguanosine; NcRNAs, non-coding RNAs; METTL3, methyltransferase-like 3; METTL14, methyltransferase-like 14; METTL16, methyltransferase-like 16; YTHDF1/2/3, YTH *N* ^6^-methyladenosine RNA binding protein 1/2/3; EMT, epithelial mesenchymal transformation; FTO, fat mass and obesity-associated protein; ALKBH5, alkylation repair homing protein 5; AML, acute myeloid leukemia; CSCs, cancer stem cells; SOX2, Sex determining region Y-box 2;NSCLC, non-small-cell lung cancer; EOC, epithelial ovarian cancer; HBXIP, hepatitis B x-interacting protein; DGCR8, DiGeorge syndrome chromosomal region 8;CarRNAs, chromatin regulatory RNAs; GSCs, glioblastoma stem cells; SOCS2, Suppressor of cytokine signal 2;YTHDC1, YTH domain-containing 1;ZC3H13, Zinc finger CCCH domain containing protein 13; RBM15, RNA-binding motif protein 15;WTAP, Wilms tumor-associated protein; MiRNA, microRNA; HuR, Human antigen R;FOXM1, Forkhead box protein M1; eIF3b, eukaryotic initiation factor 3b; GLUT4, Glucose transporter-4; HUVEC, human umbilical vein endothelial cell; EpCAM, epithelial cell adhesion molecule; ALDH, aldehyde dehydrogenase; CCND1, cyclin D1.

## References

[1] Traube FR Carell T. The Chemistries and Consequences of DNA and RNA Methylation and Demethylation. RNA Biol (2017) 14(9):1099–107. 10.1080/15476286.2017.1318241 PMC5699545 28440690

[2] Pan Y Ma P Liu Y Li W Shu Y. Multiple Functions of M(6)a RNA Methylation in Cancer. J. Hematol Oncol (2018) 11(1):48. 10.1186/s13045-018-0590-8 PMC5870302 29587823

[3] Peer E Rechavi G Dominissini D. Epitranscriptomics: Regulation of mRNA Metabolism Through Modifications. Curr Opin Chem Biol (2017) 41:93–8. 10.1016/j.cbpa.2017.10.008 29125941

[4] Patil DP Chen CK Pickering BF Chow A Jackson C Guttman M. M(6)a RNA Methylation Promotes XIST-Mediated Transcriptional Repression. Nature (2016) 537(7620):369–73. 10.1038/nature19342 PMC5509218 27602518

[5] Xiao S Cao S Huang Q Xia L Deng M Yang M. The RNA N-Methyladenosine Modification Landscape of Human Fetal Tissues. Nat Cell Biol (2019) 21(5):651–61. 10.1038/s41556-019-0315-4 31036937

[6] Blow MJ Clark TA Daum CG Deutschbauer AM Fomenkov A Fries R. The Epigenomic Landscape of Prokaryotes. PloS Genet (2016) 12(2):e1005854. 10.1371/journal.pgen.1005854 26870957 PMC4752239

[7] Gokhale NS McIntyre ABR Mattocks MD Holley CL Lazear HM Mason CE. Altered Ma Modification of Specific Cellular Transcripts Affects Flaviviridae Infection. Mol Cell (2020) 77(3):542–55. 10.1016/j.molcel.2019.11.007 PMC7007864 31810760

[8] Zhang Z Xing Y. CLIP-Seq Analysis of Multi-Mapped Reads Discovers Novel Functional RNA Regulatory Sites in the Human Transcriptome. Nucleic Acids Res (2017) 45(16):9260–71. 10.1093/nar/gkx646 PMC5766199 28934506

[9] Wu Y Xie L Wang M Xiong Q Guo Y Liang Y. Mettl3-mediated Ma RNA Methylation Regulates the Fate of Bone Marrow Mesenchymal Stem Cells and Osteoporosis. Nat Commun (2018) 9(1):4772. 10.1038/s41467-018-06898-4 30429466 PMC6235890

[10] Chen J Zhang Y Huang C Shen H Sun B Cheng X. Ma Regulates Neurogenesis and Neuronal Development by Modulating Histone Methyltransferase Ezh2. Genomics Proteomics Bioinf (2019) 17(2):154–68. 10.1016/j.gpb.2018.12.007 PMC6620265 31154015

[11] Li H Tong J Zhu S Batista P Duffy E Zhao J. M6a mRNA Methylation Controls T Cell Homeostasis by Targeting the IL-7/STAT5/SOCS Pathways. Nature (2017) 548(7667):338–42. 10.1038/nature23450 PMC5729908 28792938

[12] Batista P. The RNA Modification N6-methyladenosine and Its Implications in Human Disease. Genomics Proteomics Bioinf (2017) 15(3):154–63. 10.1016/j.gpb.2017.03.002 PMC5487527 28533023

[13] Wang S Chai P Jia R Jia R. Novel Insights on M6a RNA Methylation in Tumorigenesis: A Double-Edged Sword. Mol Cancer (2018) 17(1):101. 10.1186/s12943-018-0847-4 30031372 PMC6054842

[14] Byszewska M Śmietański M Purta E Bujnicki JM. RNA Methyltransferases Involved in 5' Cap Biosynthesis. RNA Biol (2014) 11(12):1597–607. 10.1080/15476286.2015.1004955 PMC4615557 25626080

[15] Huang H Weng H Chen J. The Biogenesis and Precise Control of RNA m6A Methylation. Trends Genet TIG (2020) 36(1):44–52. 10.1016/j.tig.2019.10.011 31810533 PMC6925345

[16] Wang X He C. Reading RNA Methylation Codes Through Methyl-Specific Binding Proteins. RNA Biol (2014) 11(6):669–72. 10.4161/rna.28829 PMC4156498 24823649

[17] Wu Y Yang X Chen Z Tian L Jiang G Chen F. M(6)a-Induced Lncrna RP11 Triggers the Dissemination of Colorectal Cancer Cells Via Upregulation of Zeb1. Mol Cancer (2019) 18(1):87. 10.1186/s12943-019-1014-2 30979372 PMC6461827

[18] Zhang C Huang S Zhuang H Ruan S Zhou Z Huang K. YTHDF2 Promotes the Liver Cancer Stem Cell Phenotype and Cancer Metastasis by Regulating OCT4 Expression Via M6a RNA Methylation. Oncogene (2020) 39(23):4507–18. 10.1038/s41388-020-1303-7 32366907

[19] Li T Hu PS Zuo Z Lin JF Li X Wu QN. METTL3 Facilitates Tumor Progression Via an M(6)a-IGF2BP2-Dependent Mechanism in Colorectal Carcinoma. Mol Cancer (2019) 18(1):112. 10.1186/s12943-019-1038-7 31230592 PMC6589893

[20] Wang Q Chen C Ding Q Zhao Y Wang Z Chen J. METTL3-Mediated M(6)a Modification of HDGF mRNA Promotes Gastric Cancer Progression and has Prognostic Significance. Gut (2020) 69(7):1193–205. 10.1136/gutjnl-2019-319639 31582403

[21] Chen M Wei L Law CT Tsang FH Shen J Cheng CL. Rna N6-methyladenosine Methyltransferase-Like 3 Promotes Liver Cancer Progression Through YTHDF2-dependent Posttranscriptional Silencing of SOCS2. Hepatology (2018) 67(6):2254–70. 10.1002/hep.29683 29171881

[22] Zhang Z Liu X Feng B Liu N Wu Q Han Y. STIM1, a Direct Target of microRNA-185, Promotes Tumor Metastasis and Is Associated With Poor Prognosis in Colorectal Cancer. Oncogene (2016) 35(46):6043. 10.1038/onc.2016.140 27375024 PMC5116556

[23] Li T Xie J Shen C Cheng D Shi Y Wu Z. Upregulation of Long Noncoding RNA Zeb1-AS1 Promotes Tumor Metastasis and Predicts Poor Prognosis in Hepatocellular Carcinoma. Oncogene (2016) 35(12):1575–84. 10.1038/onc.2015.223 26073087

[24] Nakao M Yoshida J Ishii G Hishida T Nishimura M Nagai K. Prognostic Impact of Node Involvement Pattern in Pulmonary pN1 Squamous Cell Carcinoma Patients. J Thoracic Oncol (2010) 5(4):504–9. 10.1097/JTO.0b013e3181ccb391 20104192

[25] Brown M Assen FP Leithner A Abe J Schachner H Asfour G. Lymph Node Blood Vessels Provide Exit Routes for Metastatic Tumor Cell Dissemination in Mice. Sci (New York NY) (2018) 359(6382):1408–11. 10.1126/science.aal3662 29567714

[26] Shih DJH Nayyar N Bihun I Dagogo-Jack I Gill CM Aquilanti E. Genomic Characterization of Human Brain Metastases Identifies Drivers of Metastatic Lung Adenocarcinoma. Nat Genet (2020) 52(4):371–7. 10.1038/s41588-020-0592-7 PMC7136154 32203465

[27] Liu Y Cao X. Organotropic Metastasis: Role of Tumor Exosomes. Cell Res (2015) 26(2):149–50. 10.1038/cr.2015.153 PMC4746605 26704450

[28] Mlecnik B Bindea G Kirilovsky A Angell H Obenauf A Tosolini M. The Tumor Microenvironment and Immunoscore Are Critical Determinants of Dissemination to Distant Metastasis. Sci Trans Med (2016) 8(327):327ra26. 10.1126/scitranslmed.aad6352 26912905

[29] Angelova M Mlecnik B Vasaturo A Bindea G Fredriksen T Lafontaine L. Evolution of Metastases in Space and Time Under Immune Selection. Cell (2018) 175(3):751–65.e16. 10.1016/j.cell.2018.09.018 30318143

[30] Tuncel G Kalkan R. Importance of M N6-methyladenosine (M6a) RNA Modification in Cancer. Med Oncol (2019) 36(4):36. 10.1007/s12032-019-1260-6 30879160

[31] Zhao W Qi X Liu L Ma S Liu J Wu J. Epigenetic Regulation of Ma Modifications in Human Cancer. Mol Ther Nucleic Acids (2019) 19:405–12. 10.1016/j.omtn.2019.11.022 PMC6938965 31887551

[32] Janin M Ortiz-Barahona V de Moura MC Martínez-Cardús A Llinàs-Arias P Soler M. Epigenetic Loss of RNA-methyltransferase NSUN5 in Glioma Targets Ribosomes to Drive a Stress Adaptive Translational Program. Acta Neuropathol (2019) 138(6):1053–74. 10.1007/s00401-019-02062-4 PMC6851045 31428936

[33] Niu Y Lin Z Wan A Chen H Liang H Sun L. Rna N6-methyladenosine Demethylase FTO Promotes Breast Tumor Progression Through Inhibiting BNIP3. Mol Cancer (2019) 18(1):46. 10.1186/s12943-019-1004-4 30922314 PMC6437932

[34] Liu J Jiang J Mo J Liu D Cao D Wang H. Global DNA 5-Hydroxymethylcytosine and 5-Formylcytosine Contents Are Decreased in the Early Stage of Hepatocellular Carcinoma. Hepatol (Baltimore Md) (2019) 69(1):196–208. 10.1002/hep.30146 30070373

[35] Deng R Shen N Yang Y Yu H Xu S Yang YW. Targeting Epigenetic Pathway With Gold Nanoparticles for Acute Myeloid Leukemia Therapy. Biomaterials (2018) 167:80–90. 10.1016/j.biomaterials.2018.03.013 29554483

[36] Shi H Wei J He C. Where, When, and How: Context-Dependent Functions of RNA Methylation Writers, Readers, and Erasers. Mol Cell (2019) 74(4):640–50. 10.1016/j.molcel.2019.04.025 PMC6527355 31100245

[37] McIntyre ABR Alexander N Grigorev K Bezdan D Sichtig H Chiu CY. Single-Molecule Sequencing Detection of N6-Methyladenine in Microbial Reference Materials. Nat Commun (2019) 10(1):579. 10.1038/s41467-019-08289-9 30718479 PMC6362088

[38] Dai T Pu Q Guo Y Zuo C Bai S Yang Y. Analogous Modified DNA Probe and Immune Competition Method-Based Electrochemical Biosensor for RNA Modification. Biosens Bioelectron (2018) 114:72–7. 10.1016/j.bios.2018.05.018 29783144

[39] Wang H Yin H Huang H Li K Zhou Y Waterhouse GIN. Dual-Signal Amplified Photoelectrochemical Biosensor for Detection of N(6)-Methyladenosine Based on BiVO(4)-110-TiO(2) Heterojunction, Ag(+)-Mediated Cytosine Pairs. Biosens Bioelectron (2018) 108:89–96. 10.1016/j.bios.2018.02.056 29522904

[40] Yin H Wang H Jiang W Zhou Y Ai S. Electrochemical Immunosensor for N6-methyladenosine Detection in Human Cell Lines Based on Biotin-Streptavidin System and Silver-SiO(2) Signal Amplification. Biosens Bioelectron (2017) 90:494–500. 10.1016/j.bios.2016.10.066 27825887

[41] Yang S Wei J Cui YH Park G Shah P Deng Y. M6a mRNA Demethylase FTO Regulates Melanoma Tumorigenicity and Response to anti-PD-1 Blockade. Nat Commun (2019) 10(1):2782. 10.1038/s41467-019-10669-0 31239444 PMC6592937

[42] Winkler R Gillis E Lasman L Safra M Geula S Soyris C. M6A Modification Controls the Innate Immune Response to Infection by Targeting Type I Interferons. Nat Immunol (2019) 20(2):173–82. 10.1038/s41590-018-0275-z 30559377

[43] Warda AS Kretschmer J Hackert P Lenz C Urlaub H Höbartner C. Human METTL16 is a N6-methyladenosine (m6A) Methyltransferase That Targets pre-mRNAs and Various Non-Coding RNAs. EMBO Rep (2017) 18(11):2004–14. 10.15252/embr.201744940 PMC5666602 29051200

[44] Batista PJ Molinie B Wang J Qu K Zhang J Li L. M(6)a RNA Modification Controls Cell Fate Transition in Mammalian Embryonic Stem Cells. Cell Stem Cell (2014) 15(6):707–19. 10.1016/j.stem.2014.09.019 PMC4278749 25456834

[45] Yang F Jin H Que B Chao Y Zhang H Ying X. Dynamic Ma mRNA Methylation Reveals the Role of METTL3-mA-CDCP1 Signaling Axis in Chemical Carcinogenesis. Oncogene (2019) 38(24):4755–72. 10.1038/s41388-019-0755-0 PMC6756049 30796352

[46] Bertero A Brown S Madrigal P Osnato A Ortmann D Yiangou L. The SMAD2/3 Interactome Reveals That TGF-*β* Controls Ma mRNA Methylation in Pluripotency. Nature (2018) 555(7695):256–9. 10.1038/nature25784 PMC5951268 29489750

[47] Wu R Liu Y Zhao Y Bi Z Yao Y Liu Q. M6A Methylation Controls Pluripotency of Porcine Induced Pluripotent Stem Cells by Targeting SOCS3/JAK2/STAT3 Pathway in a YTHDF1/YTHDF2-orchestrated Manner. Cell Death Dis (2019) 10(3):171. 10.1038/s41419-019-1417-4 30787270 PMC6382841

[48] Yu S Wang Y Jing L Claret FX Li Q Tian T. Autophagy in the "Inflammation-Carcinogenesis" Pathway of Liver and HCC Immunotherapy. Cancer Lett (2017) 411:82–9. 10.1016/j.canlet.2017.09.049 28987386

[49] Zhang C Chen Y Sun B Wang L Yang Y Ma D. M6A Modulates Haematopoietic Stem and Progenitor Cell Specification. Nature (2017) 549(7671):273–6. 10.1038/nature23883 28869969

[50] Lin Z Hsu PJ Xing X Fang J Lu Z Zou Q. Mettl3-/Mettl14-mediated Mrna N6-methyladenosine Modulates Murine Spermatogenesis. Cell Res (2017) 27(10):1216–30. 10.1038/cr.2017.117 PMC5630681 28914256

[51] Wang Q Chen C Ding Q Zhao Y Wang Z Chen J. METTL3-Mediated m6A Modification of HDGF mRNA Promotes Gastric Cancer Progression and has Prognostic Significance. Gut (2020) 69(7):1193–205. 10.1136/gutjnl-2019-319639 31582403

[52] Martin GH Park CY. Meddling With METTLs in Normal and Leukemia Stem Cells. Cell Stem Cell (2018) 22(2):139–41. 10.1016/j.stem.2018.01.013 29395048

[53] Rong ZX Li Z He JJ Liu LY Ren XX Gao J. Downregulation of Fat Mass and Obesity Associated (Fto) Promotes the Progression of Intrahepatic Cholangiocarcinoma. Front Oncol (2019) 9:369. 10.3389/fonc.2019.00369 31143705 PMC6521779

[54] Lan T Li H Zhang D Xu L Liu H Hao X. KIAA1429 Contributes to Liver Cancer Progression Through N6-Methyladenosine-Dependent Post-Transcriptional Modification of GATA3. Mol Cancer (2019) 18(1):186. 10.1186/s12943-019-1106-z 31856849 PMC6921542

[55] Ping XL Sun BF Wang L Xiao W Yang X Wang WJ. Mammalian WTAP is a Regulatory Subunit of the RNA N6-Methyladenosine Methyltransferase. Cell Res (2014) 24(2):177–89. 10.1038/cr.2014.3 PMC3915904 24407421

[56] He Y Hu H Wang Y Yuan H Lu Z Wu P. Alkbh5 Inhibits Pancreatic Cancer Motility by Decreasing Long Non-Coding RNA KCNK15-AS1 Methylation. Cell Physiol Biochem (2018) 48(2):838–46. 10.1159/000491915 30032148

[57] Wen J Lv R Ma H Shen H He C Wang J. Zc3h13 Regulates Nuclear RNA M6a Methylation and Mouse Embryonic Stem Cell Self-Renewal. Mol Cell (2018) 69(6):1028–38.e6. 10.1016/j.molcel.2018.02.015 29547716 PMC5858226

[58] Yue Y Liu J Cui X Cao J Luo G Zhang Z. VIRMA Mediates Preferential m6A mRNA Methylation in 3'UTR and Near Stop Codon and Associates With Alternative Polyadenylation. Cell Discovery (2018) 4:10. 10.1038/s41421-018-0019-0 29507755 PMC5826926

[59] Li XC Jin F Wang BY Yin XJ Hong W Tian FJ. The m6A Demethylase ALKBH5 Controls Trophoblast Invasion at the Maternal-Fetal Interface by Regulating the Stability of CYR61 Mrna. Theranostics (2019) 9(13):3853–65. 10.7150/thno.31868 PMC6587351 31281518

[60] Lin X Chai G Wu Y Li J Chen F Liu J. RNA M(6)a Methylation Regulates the Epithelial Mesenchymal Transition of Cancer Cells and Translation of Snail. Nat Commun (2019) 10(1):2065. 10.1038/s41467-019-09865-9 31061416 PMC6502834

[61] Wang X Wu R Liu Y Zhao Y Bi Z Yao Y. m6A mRNA Methylation Controls Autophagy and Adipogenesis by Targeting Atg5 and Atg7. Autophagy (2020) 16(7):1221–35. 10.1080/15548627.2019.1659617 PMC7469583 31451060

[62] Cui Q Shi H Ye P Li L Qu Q Sun G. M6a RNA Methylation Regulates the Self-Renewal and Tumorigenesis of Glioblastoma Stem Cells. Cell Rep (2017) 18(11):2622–34. 10.1016/j.celrep.2017.02.059 PMC5479356 28297667

[63] Zhang S Zhao BS Zhou A Lin K Zheng S Lu Z. M(6)a Demethylase Alkbh5 Maintains Tumorigenicity of Glioblastoma Stem-Like Cells by Sustaining Foxm1 Expression and Cell Proliferation Program. Cancer Cell (2017) 31(4):591–606 e6. 10.1016/j.ccell.2017.02.013 28344040 PMC5427719

[64] Simmons G Zmora P Gierer S Heurich A Pöhlmann S. Proteolytic Activation of the SARS-coronavirus Spike Protein: Cutting Enzymes at the Cutting Edge of Antiviral Research. Antiviral Res (2013) 100(3):605–14. 10.1016/j.antiviral.2013.09.028 PMC3889862 24121034

[65] Zhang C Samanta D Lu H Bullen J Zhang H Chen I. Hypoxia Induces the Breast Cancer Stem Cell Phenotype by HIF-dependent and ALKBH5-mediated M⁶a-Demethylation of NANOG mRNA. Proc Natl Acad Sci USA (2016) 113(14):E2047–56. 10.1073/pnas.1602883113 PMC4833258 27001847

[66] Zheng G Dahl JA Niu Y Fu Y Klungland A Yang YG. Sprouts of RNA Epigenetics: The Discovery of Mammalian RNA Demethylases. RNA Biol (2013) 10(6):915–8. 10.4161/rna.24711 PMC3904589 23619745

[67] Hsu PJ Shi H Zhu AC Lu Z Miller N Edens BM. The RNA-binding Protein FMRP Facilitates the Nuclear Export of N6-Methyladenosine-containing Mrnas. J Biol Chem (2019) 294(52):19889–95. 10.1074/jbc.AC119.010078 PMC6937581 31753916

[68] Yao Y Bi Z Wu R Zhao Y Liu Y Liu Q. METTL3 Inhibits BMSC Adipogenic Differentiation by Targeting the JAK1/STAT5/C/Ebpβ Pathway Via an M6a-YTHDF2-dependent Manner. FASEB J (2019) 33(6):7529–44. 10.1096/fj.201802644R 30865855

[69] Theler D Dominguez C Blatter M Boudet J Allain FH. Solution Structure of the YTH Domain in Complex With N6-methyladenosine RNA: A Reader of Methylated RNA. Nucleic Acids Res (2014) 42(22):13911–9. 10.1093/nar/gku1116 PMC4267619 25389274

[70] Sun Z Ou C Liu J Chen C Zhou Q Yang S. YAP1-Induced MALAT1 Promotes Epithelial-Mesenchymal Transition and Angiogenesis by Sponging miR-126-5p in Colorectal Cancer. Oncogene (2019) 38(14):2627–44. 10.1038/s41388-018-0628-y PMC6484768 30531836

[71] Huang H Weng H Zhou K Wu T Zhao BS Sun M. Histone H3 Trimethylation at Lysine 36 Guides M6a RNA Modification Co-Transcriptionally. Nature (2019) 567(7748):414–9. 10.1038/s41586-019-1016-7 PMC6438714 30867593

[72] Livneh I Moshitch-Moshkovitz S Amariglio N Rechavi G Dominissini D. The m6A Epitranscriptome: Transcriptome Plasticity in Brain Development and Function. Nat Rev Neurosci (2020) 21(1):36–51. 10.1038/s41583-019-0244-z 31804615

[73] Berulava T Buchholz E Elerdashvili V Pena T Islam MR Lbik D. Changes in M6a RNA Methylation Contribute to Heart Failure Progression by Modulating Translation. Eur J Heart Failure (2020) 22(1):54–66. 10.1002/ejhf.1672 31849158

[74] Dorn LE Lasman L Chen J Xu X Hund TJ Medvedovic M. The N6-Methyladenosine Mrna Methylase Mettl3 Controls Cardiac Homeostasis and Hypertrophy. Circulation (2019) 139(4):533–45. 10.1161/circulationaha.118.036146 PMC6340720 30586742

[75] Shi H Zhang X Weng YL Lu Z Liu Y Lu Z. M6A Facilitates Hippocampus-Dependent Learning and Memory Through YTHDF1. Nature (2018) 563(7730):249–53. 10.1038/s41586-018-0666-1 PMC6226095 30401835

[76] Weng H Huang H Wu H Qin X Zhao BS Dong L. Mettl14 Inhibits Hematopoietic Stem/Progenitor Differentiation and Promotes Leukemogenesis Via mRNA M(6)a Modification. Cell Stem Cell (2018) 22(2):191–205 e9. 10.1016/j.stem.2017.11.016 29290617 PMC5860916

[77] Strilic B Yang L Albarrán-Juárez J Wachsmuth L Han K Müller U. Tumour-Cell-Induced Endothelial Cell Necroptosis Via Death Receptor 6 Promotes Metastasis. Nature (2016) 536(7615):215–8. 10.1038/nature19076 27487218

[78] Okimoto R Breitenbuecher F Olivas V Wu W Gini B Hofree M. Inactivation of Capicua Drives Cancer Metastasis. Nat Genet (2017) 49(1):87–96. 10.1038/ng.3728 27869830 PMC5195898

[79] El-Naggar A Veinotte C Cheng H Grunewald T Negri G Somasekharan S. Translational Activation of HIF1α by YB-1 Promotes Sarcoma Metastasis. Cancer Cell (2015) 27(5):682–97. 10.1016/j.ccell.2015.04.003 25965573

[80] Jiang Q Crews LA Holm F Jamieson CHM. RNA Editing-Dependent Epitranscriptome Diversity in Cancer Stem Cells. Nat Rev Cancer (2017) 17(6):381–92. 10.1038/nrc.2017.23 PMC5665169 28416802

[81] Lin Y-H Wu M-H Huang Y-H Yeh C-T Cheng M-L Chi H-C. Taurine Up-Regulated Gene 1 Functions as a Master Regulator to Coordinate Glycolysis and Metastasis in Hepatocellular Carcinoma. Hepatology (2018) 67(1):188–203. 10.1002/hep.29462 28802060

[82] Liu F Ma F Wang Y Hao L Zeng H Jia C. PKM2 Methylation by CARM1 Activates Aerobic Glycolysis to Promote Tumorigenesis. Nat Cell Biol (2017) 19(11):1358–70. 10.1038/ncb3630 PMC5683091 29058718

[83] Li W Tanikawa T Kryczek I Xia H Li G Wu K. Aerobic Glycolysis Controls Myeloid-Derived Suppressor Cells and Tumor Immunity Via a Specific Cebpb Isoform in Triple-Negative Breast Cancer. Cell Metab (2018) 28(1):87–103. 10.1016/j.cmet.2018.04.022 29805099 PMC6238219

[84] Liu J Eckert MA Harada BT Liu SM Lu Z Yu K. M(6)a mRNA Methylation Regulates AKT Activity to Promote the Proliferation and Tumorigenicity of Endometrial Cancer. Nat Cell Biol (2018) 20(9):1074–83. 10.1038/s41556-018-0174-4 PMC6245953 30154548

[85] Su R Dong L Li C Nachtergaele S Wunderlich M Qing Y. R-2hg Exhibits Anti-Tumor Activity by Targeting FTO/M(6)a/MYC/CEBPA Signaling. Cell (2018) 172(1-2):90–105.e23. 10.1016/j.cell.2017.11.031 29249359 PMC5766423

[86] Xie JW Huang XB Chen QY Ma YB Zhao YJ Liu LC. M(6)a Modification-Mediated BATF2 Acts as a Tumor Suppressor in Gastric Cancer Through Inhibition of ERK Signaling. Mol Cancer (2020) 19(1):114. 10.1186/s12943-020-01223-4 32650804 PMC7350710

[87] Guo X Li K Jiang W Hu Y Xiao W Huang Y. RNA Demethylase ALKBH5 Prevents Pancreatic Cancer Progression by Posttranscriptional Activation of PER1 in an M6a-YTHDF2-Dependent Manner. Mol Cancer (2020) 19(1):91. 10.1186/s12943-020-01158-w 32429928 PMC7236181

[88] Chen X Xu M Xu X Zeng K Liu X Pan B. METTL14-Mediated N6-methyladenosine Modification of SOX4 mRNA Inhibits Tumor Metastasis in Colorectal Cancer. Mol Cancer (2020) 19(1):106. 10.1186/s12943-020-01220-7 32552762 PMC7298962

[89] Wang J Ding W Xu Y Tao E Mo M Xu W. Long non-Coding RNA Rhpn1-AS1 Promotes Tumorigenesis and Metastasis of Ovarian Cancer by Acting as a ceRNA Against miR-596 and Upregulating LETM1. Aging (2020) 12(5):4558–72. 10.18632/aging.102911 PMC7093190 32163372

[90] Peng W Li J Chen R Gu Q Yang P Qian W. Upregulated METTL3 Promotes Metastasis of Colorectal Cancer Via miR-1246/SPRED2/MAPK Signaling Pathway. J Exp Clin Cancer Res (2019) 38(1):393. 10.1186/s13046-019-1408-4 31492150 PMC6729001

[91] Jin D Guo J Wu Y Du J Yang L Wang X. M(6)a mRNA Methylation Initiated by METTL3 Directly Promotes YAP Translation and Increases YAP Activity by Regulating the MALAT1-miR-1914-3p-YAP Axis to Induce NSCLC Drug Resistance and Metastasis. J Hematol Oncol (2019) 12(1):135. 10.1186/s13045-019-0830-6 31818312 PMC6902496

[92] Ma JZ Yang F Zhou CC Liu F Yuan JH Wang F. METTL14 Suppresses the Metastatic Potential of Hepatocellular Carcinoma by Modulating N(6)-methyladenosine-dependent Primary MicroRNA Processing. Hepatology (2017) 65(2):529–43. 10.1002/hep.28885 27774652

[93] Jin D Guo J Wu Y Yang L Wang X Du J. M(6)a Demethylase ALKBH5 Inhibits Tumor Growth and Metastasis by Reducing YTHDFs-mediated YAP Expression and Inhibiting Mir-107/LATS2-Mediated YAP Activity in NSCLC. Mol Cancer (2020) 19(1):40. 10.1186/s12943-020-01161-1 32106857 PMC7045432

[94] Yang J Antin P Berx G Blanpain C Brabletz T Bronner M. Guidelines and Definitions for Research on Epithelial-Mesenchymal Transition. Nat Rev Mol Cell Biol (2020) 21(6):341–52. 10.1038/s41580-020-0237-9 PMC7250738 32300252

[95] Jung HY Fattet L Tsai JH Kajimoto T Chang Q Newton AC. Apical-Basal Polarity Inhibits Epithelial-Mesenchymal Transition and Tumour Metastasis by PAR-Complex-Mediated SNAI1 Degradation. Nat Cell Biol (2019) 21(3):359–71. 10.1038/s41556-019-0291-8 PMC6546105 30804505

[96] Basu S Barad M Yadav D Nandy A Mukherjee B Sarkar J. DBC1, P300, HDAC3, and Siah1 Coordinately Regulate ELL Stability and Function for Expression of its Target Genes. Proc Natl Acad Sci USA (2020) 117(12):6509–20. 10.1073/pnas.1912375117 PMC7104407 32152128

[97] Díaz V de Herreros A. F-Box Proteins: Keeping the Epithelial-to-Mesenchymal Transition (EMT) in Check. Semin Cancer Biol (2016) 36:71–9. 10.1016/j.semcancer.2015.10.003 26506454

[98] Title A Hong S Pires N Hasenöhrl L Godbersen S Stokar-Regenscheit N. Genetic Dissection of the miR-200-Zeb1 Axis Reveals Its Importance in Tumor Differentiation and Invasion. Nat Commun (2018) 9(1):4671. 10.1038/s41467-018-07130-z 30405106 PMC6220299

[99] DeBerardinis RJ Chandel NS. Fundamentals of Cancer Metabolism. Sci Adv (2016) 2(5):e1600200. 10.1126/sciadv.1600200 27386546 PMC4928883

[100] Meng F Wu L Dong L Mitchell AV James Block C Liu J. EGFL9 Promotes Breast Cancer Metastasis by Inducing cMET Activation and Metabolic Reprogramming. Nat Commun (2019) 10(1):5033. 10.1038/s41467-019-13034-3 31695034 PMC6834558

[101] Wang Y Zhang X Wang Z Hu Q Wu J Li Y. LncRNA-p23154 Promotes the Invasion-Metastasis Potential of Oral Squamous Cell Carcinoma by Regulating Glut1-Mediated Glycolysis. Cancer Lett (2018) 434:172–83. 10.1016/j.canlet.2018.07.016 30026052

[102] Mao L Dauchy RT Blask DE Dauchy EM Slakey LM Brimer S. Melatonin Suppression of Aerobic Glycolysis (Warburg Effect), Survival Signalling and Metastasis in Human Leiomyosarcoma. J Pineal Res (2016) 60(2):167–77. 10.1111/jpi.12298 26607298

[103] Chang YC Chi LH Chang WM Su CY Lin YF Chen CL. Glucose Transporter 4 Promotes Head and Neck Squamous Cell Carcinoma Metastasis Through the TRIM24-DDX58 Axis. J Hematol Oncol (2017) 10(1):11. 10.1186/s13045-016-0372-0 28061796 PMC5219690

[104] Zheng Y Wu C Yang J Zhao Y Jia H Xue M. Insulin-Like Growth Factor 1-Induced Enolase 2 Deacetylation by HDAC3 Promotes Metastasis of Pancreatic Cancer. Signal Transduct Target Ther (2020) 5(1):53. 10.1038/s41392-020-0146-6 32398667 PMC7217878

[105] Roh W Chen PL Reuben A Spencer CN Prieto PA Miller JP. Integrated Molecular Analysis of Tumor Biopsies on Sequential CTLA-4 and PD-1 Blockade Reveals Markers of Response and Resistance. Sci Transl Med (2017) 9(379):eaah3560. 10.1126/scitranslmed.aah3560 28251903 PMC5819607

[106] Shen R Li P Li B Zhang B Feng L Cheng S. Identification of Distinct Immune Subtypes in Colorectal Cancer Based on the Stromal Compartment. Front Oncol (2019) 9:1497. 10.3389/fonc.2019.01497 31998649 PMC6965328

[107] Kuczynski E Vermeulen P Pezzella F Kerbel R Reynolds A. Vessel Co-Option in Cancer. Nat Rev Clin Oncol (2019) 16(8):469–93. 10.1038/s41571-019-0181-9 30816337

[108] Donnem T Reynolds A Kuczynski E Gatter K Vermeulen P Kerbel R. Non-Angiogenic Tumours and Their Influence on Cancer Biology. Nat Rev Cancer (2018) 18(5):323–36. 10.1038/nrc.2018.14 29520090

[109] Matsubara T Kanto T Kuroda S Yoshio S Higashitani K Kakita N. TIE2-Expressing Monocytes as a Diagnostic Marker for Hepatocellular Carcinoma Correlates With Angiogenesis. Hepatol (Baltimore Md) (2013) 57(4):1416–25. 10.1002/hep.25965 22815256

[110] Jordan C Guzman M Noble M. Cancer Stem Cells. New Engl J Med (2006) 355(12):1253–61. 10.1056/NEJMra061808 16990388

[111] Saygin C Matei D Majeti R Reizes O Lathia JD. Targeting Cancer Stemness in the Clinic: From Hype to Hope. Cell Stem Cell (2019) 24(1):25–40. 10.1016/j.stem.2018.11.017 30595497

[112] Boumahdi S Driessens G Lapouge G Rorive S Nassar D Le Mercier M. SOX2 Controls Tumour Initiation and Cancer Stem-Cell Functions in Squamous-Cell Carcinoma. Nature (2014) 511(7508):246–50. 10.1038/nature13305 24909994

[113] Lu H Xie Y Tran L Lan J Yang Y Murugan N. Chemotherapy-Induced S100A10 Recruits KDM6A to Facilitate OCT4-mediated Breast Cancer Stemness. J Clin Invest (2020) 130(9):4607–23. 10.1172/jci138577 PMC7456215 32427586

[114] Wang X Jin J Wan F Zhao L Chu H Chen C. Ampk Promotes Spop-Mediated NANOG Degradation to Regulate Prostate Cancer Cell Stemness. Dev Cell (2019) 48(3):345–60.e7. 10.1016/j.devcel.2018.11.033 30595535 PMC7523188

[115] Dontu G Abdallah W Foley J Jackson K Clarke M Kawamura M. In Vitro Propagation and Transcriptional Profiling of Human Mammary Stem/Progenitor Cells. Genes Dev (2003) 17(10):1253–70. 10.1101/gad.1061803 PMC196056 12756227

[116] Ginestier C Hur M Charafe-Jauffret E Monville F Dutcher J Brown M. ALDH1 is a Marker of Normal and Malignant Human Mammary Stem Cells and a Predictor of Poor Clinical Outcome. Cell Stem Cell (2007) 1(5):555–67. 10.1016/j.stem.2007.08.014 PMC2423808 18371393

[117] Xu Z Zeng X Xu J Xu D Li J Jin H. Isorhapontigenin Suppresses Growth of Patient-Derived Glioblastoma Spheres Through Regulating Mir-145/SOX2/Cyclin D1 Axis. Neuro-oncology (2016) 18(6):830–9. 10.1093/neuonc/nov298 PMC4864260 26681767

[118] Park S Seo K So A Seo M Yu K Kang S. SOX2 has a Crucial Role in the Lineage Determination and Proliferation of Mesenchymal Stem Cells Through Dickkopf-1 and C-MYC. Cell Death Differ (2012) 19(3):534–45. 10.1038/cdd.2011.137 PMC3278737 22015605

[119] Xu C Xie D Yu S Yang X He L Yang J. *β*-Catenin/POU5F1/SOX2 Transcription Factor Complex Mediates IGF-I Receptor Signaling and Predicts Poor Prognosis in Lung Adenocarcinoma. Cancer Res (2013) 73(10):3181–9. 10.1158/0008-5472.Can-12-4403 23539445

[120] Li CF Chen JY Ho YH Hsu WH Wu LC Lan HY. Snail-Induced claudin-11 Prompts Collective Migration for Tumour Progression. Nat Cell Biol (2019) 21(2):251–62. 10.1038/s41556-018-0268-z 30664792

[121] Zheng W Flavell R. The Transcription Factor GATA-3 is Necessary and Sufficient for Th2 Cytokine Gene Expression in CD4 T Cells. Cell (1997) 89(4):587–96. 10.1016/s0092-8674(00)80240-8 9160750

[122] Chan P Han X Zheng B DeRan M Yu J Jarugumilli G. Autopalmitoylation of TEAD Proteins Regulates Transcriptional Output of the Hippo Pathway. Nat Chem Biol (2016) 12(4):282–9. 10.1038/nchembio.2036 PMC4798901 26900866

[123] Li Z Weng H Su R Weng X Zuo Z Li C. Fto Plays an Oncogenic Role in Acute Myeloid Leukemia as a N6-Methyladenosine Rna Demethylase. Cancer Cell (2017) 31(1):127–41. 10.1016/j.ccell.2016.11.017 PMC5234852 28017614

[124] Liu J Dou X Chen C Chen C Liu C Xu MM. N6-Methyladenosine of Chromosome-Associated Regulatory RNA Regulates Chromatin State and Transcription. Sci (New York NY) (2020) 367(6477):580–6. 10.1126/science.aay6018 PMC7213019 31949099

[125] Satpathy A Chang H. Long Noncoding RNA in Hematopoiesis and Immunity. Immunity (2015) 42(5):792–804. 10.1016/j.immuni.2015.05.004 25992856

[126] Yang L Lin C Liu W Zhang J Ohgi K Grinstein J. ncRNA- and Pc2 Methylation-Dependent Gene Relocation Between Nuclear Structures Mediates Gene Activation Programs. Cell (2011) 147(4):773–88. 10.1016/j.cell.2011.08.054 PMC3297197 22078878

[127] Cech T Steitz J. The Noncoding RNA Revolution-Trashing Old Rules to Forge New Ones. Cell (2014) 157(1):77–94. 10.1016/j.cell.2014.03.008 24679528

[128] Chaib I Karachaliou N Pilotto S Codony Servat J Cai X Li X. Co-Activation of STAT3 and YES-Associated Protein 1 (Yap1) Pathway in EGFR-Mutant Nsclc. J Natl Cancer Inst (2017) 109(9):djx014. 10.1093/jnci/djx014 PMC5409000 28376152

[129] Omori H Nishio M Masuda M Miyachi Y Ueda F Nakano T. YAP1 is a Potent Driver of the Onset and Progression of Oral Squamous Cell Carcinoma. Sci Adv (2020) 6(12):eaay3324. 10.1126/sciadv.aay3324 32206709 PMC7080500

[130] Lopez-Bertoni H Lal B Li A Caplan M Guerrero-Cázares H Eberhart CG. DNMT-Dependent Suppression of microRNA Regulates the Induction of GBM Tumor-Propagating Phenotype by Oct4 and Sox2. Oncogene (2015) 34(30):3994–4004. 10.1038/onc.2014.334 25328136 PMC4404208

[131] Luzzi A Morettini F Gazaneo S Mundo L Onnis A Mannucci S. Hiv-1 Tat Induces DNMT Over-Expression Through microRNA Dysregulation in HIV-related non Hodgkin Lymphomas. Infect Agents Cancer (2014) 9:41. 10.1186/1750-9378-9-41 PMC4334912 25705251

[132] Braga EA Fridman MV Moscovtsev AA Filippova EA Dmitriev AA Kushlinskii NE. LncRNAs in Ovarian Cancer Progression, Metastasis, and Main Pathways: ceRNA and Alternative Mechanisms. Int J Mol Sci (2020) 21(22):8855. 10.3390/ijms21228855 PMC7700431 33238475

[133] Inamura K. Colorectal Cancers: An Update on Their Molecular Pathology. Cancers (Basel) (2018) 10(1):26. 10.3390/cancers10010026 PMC5789376 29361689

[134] Ricci-Vitiani L Lombardi DG Pilozzi E Biffoni M Todaro M Peschle C. Identification and Expansion of Human Colon-Cancer-Initiating Cells. Nature (2007) 445(7123):111–5. 10.1038/nature05384 17122771

[135] Ju HQ Lu YX Chen DL Tian T Mo HY Wei XL. Redox Regulation of Stem-like Cells Though the CD44v-xCT Axis in Colorectal Cancer: Mechanisms and Therapeutic Implications. Theranostics (2016) 6(8):1160–75. 10.7150/thno.14848 PMC4893643 27279909

[136] Forner A Llovet JM Bruix J. Hepatocellular Carcinoma. Lancet (2012) 3\(9822):1245–55. 10.1016/s0140-6736(11)61347-0 PMC13537732 22353262

[137] Llovet JM. Updated Treatment Approach to Hepatocellular Carcinoma. J Gastroenterol (2005) 40(3):225–35. 10.1007/s00535-005-1566-3 15830281

[138] Cabibbo G Petta S Barbara M Attardo S Bucci L Farinati F. Hepatic Decompensation is the Major Driver of Death in HCV-infected Cirrhotic Patients With Successfully Treated Early Hepatocellular Carcinoma. J Hepatol (2017) 67(1):65–71. 10.1016/j.jhep.2017.01.033 28192185

[139] Torre LA Siegel RL Ward EM Jemal A. Global Cancer Incidence and Mortality Rates and Trends–An Update. Cancer Epidemiol Biomarkers Prev (2016) 25(1):16–27. 10.1158/1055-9965.Epi-15-0578 26667886

[140] Perusina Lanfranca M Thompson JK Bednar F Halbrook C Lyssiotis C Levi B. Metabolism and Epigenetics of Pancreatic Cancer Stem Cells. Semin Cancer Biol (2019) 57:19–26. 10.1016/j.semcancer.2018.09.008 30273655 PMC6438777

[141] Paradise BD Barham W Fernandez-Zapico ME. Targeting Epigenetic Aberrations in Pancreatic Cancer, a New Path to Improve Patient Outcomes? Cancers (2018) 10(5):128. 10.3390/cancers10050128 PMC5977101 29710783

[142] Bridgewater J Galle PR Khan SA Llovet JM Park JW Patel T. Guidelines for the Diagnosis and Management of Intrahepatic Cholangiocarcinoma. J Hepatol (2014) 60(6):1268–89. 10.1016/j.jhep.2014.01.021 24681130

[143] Akram M Iqbal M Daniyal M Khan AU. Awareness and Current Knowledge of Breast Cancer. Biol Res (2017) 50(1):33. 10.1186/s40659-017-0140-9 28969709 PMC5625777

[144] Cai X Wang X Cao C Gao Y Zhang S Yang Z. HBXIP-Elevated Methyltransferase METTL3 Promotes the Progression of Breast Cancer Via Inhibiting Tumor Suppressor Let-7g. Cancer Lett (2018) 415:11–9. 10.1016/j.canlet.2017.11.018 29174803

[145] Lin S Choe J Du P Triboulet R Gregory RI. The M(6)a Methyltransferase Mettl3 Promotes Translation in Human Cancer Cells. Mol Cell (2016) 62(3):335–45. 10.1016/j.molcel.2016.03.021 PMC4860043 27117702

[146] Zhou Y Hambly BD McLachlan CS. FTO Associations With Obesity and Telomere Length. J BioMed Sci (2017) 24(1):65. 10.1186/s12929-017-0372-6 28859657 PMC5580219

[147] Wagner R Tabak AG Fehlert E Fritsche L Jaghutriz BA Banhegyi RJ. Excessive Fuel Availability Amplifies the FTO-Mediated Obesity Risk: Results From the TUEF and Whitehall II Studies. Sci Rep (2017) 7(1):15486. 10.1038/s41598-017-15744-4 29138452 PMC5686126

[148] Zhao L Ji G Le X Wang C Xu L Feng M. Long Noncoding Rna LINC00092 Acts in Cancer-Associated Fibroblasts to Drive Glycolysis and Progression of Ovarian Cancer. Cancer Res (2017) 77(6):1369–82. 10.1158/0008-5472.CAN-16-1615 28087599

[149] Wu DD Chen X Sun KX Wang LL Chen S Zhao Y. Role of the Lncrna ABHD11-AS1 in the Tumorigenesis and Progression of Epithelial Ovarian Cancer Through Targeted Regulation of Rhoc. Mol Cancer (2017) 16(1):138. 10.1186/s12943-017-0709-5 28818073 PMC5561620

[150] Mitra R Chen X Greenawalt EJ Maulik U Jiang W Zhao Z. Decoding Critical Long non-Coding RNA in Ovarian Cancer Epithelial-to-Mesenchymal Transition. Nat Commun (2017) 8(1):1604. 10.1038/s41467-017-01781-0 29150601 PMC5693921

[151] Chi AC Day TA Neville BW. Oral Cavity and Oropharyngeal Squamous Cell Carcinoma-an Update. CA: A Cancer J Clin (2015) 65(5):401–21. 10.3322/caac.21293 26215712

[152] Liu L Wu Y Li Q Liang J He Q Zhao L. Mettl3 Promotes Tumorigenesis and Metastasis Through BMI1 M(6)a Methylation in Oral Squamous Cell Carcinoma. Mol Ther (2020) 28(10):2177–90. 10.1016/j.ymthe.2020.06.024 PMC7544972 32621798

[153] Jia L Zhang W Wang C. Bmi1 Inhibition Eliminates Residual Cancer Stem Cells After PD1 Blockade and Activates Antitumor Immunity to Prevent Metastasis and Relapse. Cell Stem Cell (2020) 27(2):238–53.e6. 10.1016/j.stem.2020.06.022 32697949 PMC7416748

[154] Guo YM Chen JR Feng YC Chua MLK Zeng Y Hui EP. Germline Polymorphisms and Length of Survival of Nasopharyngeal Carcinoma: An Exome-Wide Association Study in Multiple Cohorts. Adv Sci (Weinh) (2020) 7(10):1903727. 10.1002/advs.201903727 32440486 PMC7237860

[155] Tsang CM Lui VWY Bruce JP Pugh TJ Lo KW. Translational Genomics of Nasopharyngeal Cancer. Semin Cancer Biol (2020) 61:84–100. 10.1016/j.semcancer.2019.09.006 31521748

[156] Lu S Yu Z Xiao Z Zhang Y. Gene Signatures and Prognostic Values of M(6)a Genes in Nasopharyngeal Carcinoma. Front Oncol (2020) 10:875. 10.3389/fonc.2020.00875 32596151 PMC7300221

[157] Visvanathan A Patil V Arora A Hegde AS Arivazhagan A Santosh V. Essential Role of METTL3-Mediated m6A Modification in Glioma Stem-Like Cells Maintenance and Radioresistance. Oncogene (2018) 37(4):522–33. 10.1038/onc.2017.351 28991227

[158] Jia Y Gu D Wan J Yu B Zhang X Chiorean EG. The Role of GLI-SOX2 Signaling Axis for Gemcitabine Resistance in Pancreatic Cancer. Oncogene (2019) 38(10):1764–77. 10.1038/s41388-018-0553-0 PMC6408295 30382189

[159] Huang Y Yan J Li Q Li J Gong S Zhou H. Meclofenamic Acid Selectively Inhibits FTO Demethylation of m6A Over ALKBH5. Nucleic Acids Res (2015) 43(1):373–84. 10.1093/nar/gku1276 PMC4288171 25452335

[160] Niu Y Wan A Lin Z Lu X Wan G. N(6)-Methyladenosine Modification: A Novel Pharmacological Target for Anti-Cancer Drug Development. Acta Pharm Sin B (2018) 8(6):833–43. 10.1016/j.apsb.2018.06.001 PMC6251950 30505654

